# Luminescent Alendronic Acid-Conjugated Micellar Nanostructures for Potential Application in the Bone-Targeted Delivery of Cholecalciferol

**DOI:** 10.3390/molecules29102367

**Published:** 2024-05-17

**Authors:** Federica Rizzi, Annamaria Panniello, Roberto Comparelli, Ilaria Arduino, Elisabetta Fanizza, Rosa Maria Iacobazzi, Maria Grazia Perrone, Marinella Striccoli, Maria Lucia Curri, Antonio Scilimati, Nunzio Denora, Nicoletta Depalo

**Affiliations:** 1 CNR-Institute for Chemical and Physical Process, 70125 Bari, Italy; f.rizzi@ba.ipcf.cnr.it (F.R.); a.panniello@ba.ipcf.cnr.it (A.P.); r.comparelli@ba.ipcf.cnr.it (R.C.); elisabetta.fanizza@uniba.it (E.F.); m.striccoli@ba.ipcf.cnr.it (M.S.); marialucia.curri@uniba.it (M.L.C.); 2 Consorzio Interuniversitario Nazionale per la Scienza e Tecnologia dei Materiali (INSTM), 50121 Firenze, Italy; 3 Department of Pharmacy—Pharmaceutical Sciences, University of Bari, 70125 Bari, Italy; ilaria.arduino@uniba.it (I.A.); rosa.iacobazzi@uniba.it (R.M.I.); 4 Department of Chemistry, University of Bari, 70125 Bari, Italy; 5 Research Laboratory for Woman and Child Health, Department of Pharmacy—Pharmaceutical Sciences, University of Bari, 70125 Bari, Italy; mariagrazia.perrone@uniba.it (M.G.P.); antonio.scilimati@uniba.it (A.S.)

**Keywords:** micellar nanostructures, luminescent carbon dots, cholecalciferol, alendronate, hydroxyapatite nanoparticles, active bone targeting

## Abstract

Vitamin D, an essential micronutrient crucial for skeletal integrity and various non-skeletal physiological functions, exhibits limited bioavailability and stability in vivo. This study is focused on the development of polyethylene glycol (PEG)-grafted phospholipid micellar nanostructures co-encapsulating vitamin D3 and conjugated with alendronic acid, aimed at active bone targeting. Furthermore, these nanostructures are rendered optically traceable in the UV–visible region of the electromagnetic spectrum via the simultaneous encapsulation of vitamin D3 with carbon dots, a newly emerging class of fluorescents, biocompatible nanoparticles characterized by their resistance to photobleaching and environmental friendliness, which hold promise for future in vitro bioimaging studies. A systematic investigation is conducted to optimize experimental parameters for the preparation of micellar nanostructures with an average hydrodynamic diameter below 200 nm, ensuring colloidal stability in physiological media while preserving the optical luminescent properties of the encapsulated carbon dots. Comprehensive chemical-physical characterization of these micellar nanostructures is performed employing optical and morphological techniques. Furthermore, their binding affinity for the principal inorganic constituent of bone tissue is assessed through a binding assay with hydroxyapatite nanoparticles, indicating significant potential for active bone-targeting. These formulated nanostructures hold promise for novel therapeutic interventions to address skeletal-related complications in cancer affected patients in the future.

## 1. Introduction

In the advanced stages of various types of cancers, such as breast, prostate, lung, and multiple melanoma, the occurrence of bone metastases is quite common. These bone metastases give rise to several significant skeletal-related events (SREs), which include issues such as fractures, severe pain, limited mobility, spinal compression, and hypercalcemia. These complications not only significantly affect the quality of life for oncology patients but also reduce their overall survival rate. Additionally, patients experiencing SREs require extended hospital stays, resulting in substantial healthcare costs [1].

In the context of standard clinical practice, while a multidisciplinary approach is often employed to provide the best therapeutic outcomes for bone metastasis treatments, the primary treatment option for patients with SREs is the use of anti-resorptive agents, such as bisphosphonates (BPs) [1,2]. BPs are synthetic analogues of naturally pyrophosphate compounds that inhibit bone resorption and bone formation [3]. In vitro studies have reported that BPs may have a direct action on tumour cells by inducing apoptosis, inhibiting the adhesion of tumour cells within the bone [4]. Although prolonged BP administration is generally well-tolerated by oncology patients, it can lead to the development of hypocalcaemia due to the BPs’ sustained anti-resorptive effects. Another risk factor for hypocalcaemia is vitamin D deficiency. Therefore, it is recommended to supplement with calcium and vitamin D during the BP treatment to mitigate the risk of hypocalcaemia [1,5]. Vitamin D plays crucial roles in skeletal health, including bone strength and calcium absorption, as well as in various non-skeletal functions. Proper levels of vitamin D have been associated with a reduced risk of conditions such as diabetes, cardiovascular diseases, and certain cancers [6,7,8,9]. Between the two primary forms of vitamin D, namely ergocalciferol (vitamin D2) and cholecalciferol (vitamin D3, VitD3), in humans, VitD3 is more effective than vitamin D2, primarily because it is more efficient in elevating blood serum levels of biologically active vitamin D metabolites, thanks to its higher affinity for the vitamin-D-binding protein [10]. The physiological benefits and functionality of VitD3 are constrained due to its classification as a fat-soluble micronutrient, characterized by its limited water solubility, heightened vulnerability to environmental factors, and susceptibility to oxidation. In accordance with a multitude of research studies, it was observed that a significant proportion exceeding 75% of ingested VitD3 undergoes processes of catabolism and excretion prior to its conversion into its biologically active form [11]. The clinical utilization of VitD3 is further marked by its notably brief half-life in the circulatory system and its susceptibility to the first-pass effect. Furthermore, excessive consumption of VitD3 may induce toxic effects associated with the development of hypervitaminosis syndrome [12]. To address these inherent constraints, novel drug delivery nanosystems have been designed for the formulation of VitD3, encompassing polymeric- or lipid-based nanovectors, as recently reported [11,12,13,14,15,16]. The primary objective of these nanosystems is to encapsulate VitD3, thereby not only augmenting its bioavailability and in vivo stability but also facilitating the administration of the optimal dosages, thereby mitigating the potential risks associated with toxicity.

While several studies have been conducted to explore the encapsulation of VitD3, there is a paucity of research focusing on the utilization of nanocarriers for the targeted delivery of VitD3 to the skeletal system [17]. It is important to recognize that bones are encased by lining cells that serve as a protective blood–marrow barrier, consequently limiting direct contact between bone surfaces and exogenous compounds. Furthermore, in mineralized tissues, the expression of biomolecules with specific targeting capabilities, such as enzymes or antigens, remains relatively low, thereby limiting the opportunities for active targeting. In the realm of drug delivery nanosystems tailored for bone targeting, only certain agents have emerged as the most commonly utilized. These include the arginine–glycine–aspartic acid peptide, as well as two notable members of the BP family, namely alendronic (ALE) and zoledronic acids [18,19,20]. Extensive research has revealed the substantial potential of these agents in the development of pharmaceuticals with a bone-targeted focus [21,22,23,24,25,26,27,28]. The distinctive composition of bone, characterized by high concentrations of calcium and phosphorus, contributes to its supramolecular structure, rendering it a unique and specific target for binding by BPs. The presence of hydroxyapatite (HA) during bone remodelling further enhances the affinity of mineral-binding drugs for sites exhibiting high turnover rates, often associated with pathological conditions [29]. Compounds such as BPs exploit these inherent characteristics to facilitate robust and precise in vivo binding to bone tissue [30,31]. Indeed, BPs, among which ALE is notable, possess a unique capability to chelate with calcium ions present in HA crystals, which constitute a fundamental mineral component within the bone microenvironment [32]. This distinctive property renders HA crystals ideal and specific binding sites for drugs designed for bone targeting [33].

This study aims to elucidate the use of polyethylene glycol (PEG)-grafted phospholipids in combination with luminescent carbon dots (C-dots) to fabricate optically traceable micellar nanostructures for encapsulating VitD3, which are further conjugated with ALE (VitD3/C-dot/ALE–micelles). C-dots are renowned for their intense photoluminescence (PL) characteristics in the visible range, marked by exceptional biocompatibility and robust photostability. Consequently, C-dots have extensive applications in diverse fields such as live cell imaging, biosensing, targeted drug delivery, and various biomedical applications [34]. The incorporation of functional nanoparticles (NPs) such as C-dots into the hydrophobic core of polymer-grafted lipid micelles represents an effective approach for the co-encapsulation of a wide spectrum of poorly water-soluble therapeutic agents. This approach yields multifunctional nanoformulations characterized by noteworthy colloidal stability, a high degree of biocompatibility, and stealth properties, thereby enhancing their suitability for various biomedical applications [35,36,37,38,39,40].

Here, a comprehensive characterization of the micellar nanostructures specifically designed for the bone-targeted delivery of VitD3 was conducted at each stage of the preparation process using a combination of optical and morphological techniques. The optical properties of the C-dots were investigated both before and after their integration into the micellar cores, as well as following their conjugation with ALE. This investigation aimed to evaluate the preservation of their PL, which is crucial for achieving optical traceability. This information will be valuable for future in vitro studies. Here, the PL properties of the ALE-conjugated micellar nanostructures were investigated in this study to assess their binding affinity for HA NPs, with the aim of demonstrating the robust affinity of the novel formulation for the primary inorganic component of bone. This validation underscores its potential for actively targeting bone tissue.

## 2. Results and Discussion

### 2.1. Preparation of PEGylated Phospholipid-Based Micellar Nanostructures

Oil-soluble C-dots were obtained using a one-step synthetic method as the preliminary step in the preparation of luminescent micellar nanostructures. In the synthetic process, citric acid served as the carbon precursor, while 1-octadecene (ODE) and 1-hexadecylamine (HDA) were employed as a high-boiling point solvent and surface-passivating agent, respectively [41].

**Figure 1:**
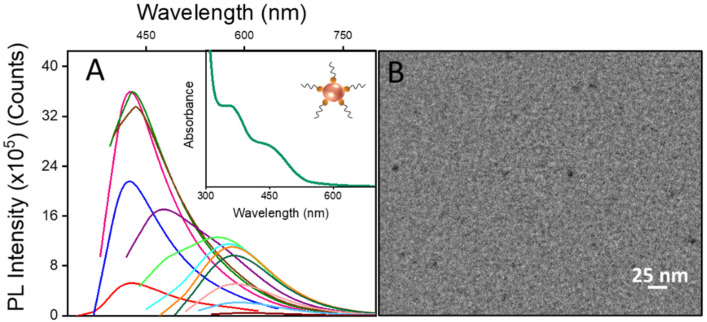
(**A**) PL spectra of the ‘as synthesized’ C-dots dispersed in organic solvent and recorded at increasing excitation wavelengths (λ) of 300 nm (black line), 320 nm (red line), 340 nm (blue line), 360 nm (magenta line), 375 nm (olive line), 380 nm (brown line), 400 nm (violet line), 420 nm (lime green line), 440 nm (light blue line), 460 nm (orange line), 480 nm (dark blue line), 500 nm (pink line), 520 nm (heavenly blue line), and 540 nm (purple line). Inset: UV-Vis absorption spectrum. (**B**) TEM micrograph of oil-soluble C-dots cast from chloroform.

The TEM analysis (Figure 1B) exhibited the formation of spherical-shaped C-dots with an average diameter of approximately 3.5 nm (σ = 23%). The UV-Vis absorption spectrum of a chloroform dispersion of ‘as synthesized’ C-dots (Figure 1A inset) displayed a distinctive peak in the range of 300 to 400 nm. This peak was attributed to the n-π* transition, which was associated with both the C=O bonds and surface chemical functional groups. Furthermore, the spectrum revealed a broadened absorption band centred around 450 nm, ascribed to the formation of molecular fluorophore aggregates during the carbonization process [41].

The PL spectroscopic measurements of the chloroform dispersion of C-dots (Figure 1A) indicated a consistent dependence of the emission band from the excitation wavelength in the visible range up to 540 nm, where the signal appeared sensibly quenched. Notably, the highest PL quantum yield (QY) of (25.3 ± 0.1)% was achieved for excitation at λ = 360 nm (Figure S1B, see Supplementary Materials (SM)).

To encapsulate oil-soluble C-dots within the hydrophobic micellar cores, an experimental procedure previously established for the preparation of PEG-stabilized micellar nanostructures that incorporate luminescent quantum dots or superparamagnetic NPs [35,36,38,39,40] was followed. Specifically, PEG-stabilized micellar nanostructures were obtained using mainly PEG-2-PE (1,2-Dipalmitoyl-sn-glycero-3-phosphoethanolamine-N-[methoxy (poly (ethylene glycol))-2000; 16:0), with only a very low percentage (molar ratio of 4:1) of the lipid mixture composed of DSPE-PEG-COOH (1,2-distearoyl-sn-glycero-3-phosphoethanolamine-N-[carboxy(polyethylene glycol)-2000), ensuring the presence of carboxylic groups on the micellar surface for conjugation with ALE. Here, PEG-2-PE-based micelles were selected as suitable nanovectors for drug delivery based on the findings of Wang et al., who demonstrated the safety of PEG-2-PE in normal cells and its toxicity in cancer cells. The same report also clarified the molecular mechanism of the cytotoxicity in cancer cells, demonstrating that PEG-2-PE micelles insert into cell membranes without compromising the membrane integrity. The micelles disassemble at the membrane level and the resulting disassembled PEG-PE molecules can be internalized into cells through non-specific endocytosis and accumulate in the endoplasmic reticulum (ER), leading to ER stress. Notably, cancer cells initiate proapoptotic signaling under PEG-2-PE-induced ER stress, while normal cells activate pro-survival signaling pathways. This suggests that PEG-2-PE micelles can safely serve as suitable nanocarriers for delivering anticancer drugs [42].

**Figure 2:**
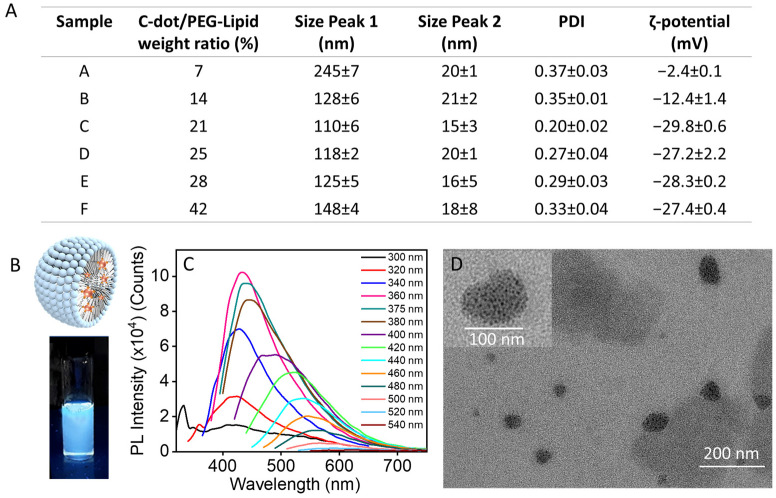
(**A**) DLS analysis and ζ-potential values for micellar nanostructures prepared at different starting C-dot/PEG–phospholipid weight ratio percentages. (**B**) Picture of the vial containing micellar nanostructures encapsulating C-Dots, acquired under 400 nm light exposure along with a representative scheme. (**C**) PL emission spectra recorded at excitation wavelengths ranging from 300 to 540 nm (1:3 dilution of sample C in PBS) and (**D**) representative TEM micrograph and corresponding close-up view in the inset cast from the water from sample C.

Different starting percentages of the C-dot/PEG–phospholipid weight ratio, ranging from 7% to 42% (samples A, B, C, D, E, and F), as indicated in Figure 2A, were systematically explored in the preparation of the luminescent PEG-stabilized micellar nanostructures. This investigation aimed to pinpoint the optimal experimental conditions to both preserve the distinctive optical characteristics of the C-dots and yield micellar aggregates with an average hydrodynamic diameter below 200 nm (Figure 2A–D) [43,44].

Prior to their characterization in terms of the dimensional, morphological, and optical properties, the ‘as-prepared’ luminescent micellar nanostructures underwent purification steps. These included centrifugation to recover the non-encapsulated oil-soluble C-dots as a pellet, followed by filtration to remove the excess lipids not assembled in the micellar structures, as described in the Section 3.

The dynamic light scattering (DLS) analysis revealed that the different samples exhibited a bimodal size distribution. Specifically, the size distribution profile exhibited a peak range centred at 15–20 nm, attributed to the formation of empty micelles, in addition to a peak due to micellar structures of a larger size (Figure 2A), encapsulating the C-dots.

The recorded average hydrodynamic diameters ranged from 110 to 150 nm for samples B–E and increased as the C-dot/phospholipid weight ratio percentage increased from 21 to 42%. Conversely, sample A, obtained at the lowest investigated C-dot/phospholipid weight ratio percentage, exhibited a larger average hydrodynamic diameter. However, the presence of a peak at sizes larger than empty micelles suggests the formation of micellar nanostructures with multiple C-dots embedded within the micellar core.

The ζ-potential analysis revealed, for all investigated samples, the presence of a negative surface charge on the PEG–micelle surface, which was decorated with carboxylic groups. It also demonstrated significant colloidal stability in a physiological medium (phosphate-buffered solution (PBS), 10 mM, pH 7.4), for samples C and D, while lower ζ-potential values were recorded for samples A and B, indicating poorer colloidal stability in aqueous environments compared to samples C and D (Figure 2A). Based on the DLS and ζ-potential measurements, samples A and B were not further considered. The PL characterization spectroscopy was performed on samples C–E. In Figure 2B,C, the UV-Vis and PL spectra of sample C are reported, while in Figure S1 of the Supplementary Materials the spectra resulting from the PL characterization of samples D and E can be observed. The findings demonstrated that despite the manipulation undergone by the C-dots in the encapsulation process into the micellar nanosystems and the considerable variation of the chemical surroundings they experienced, the peculiar optical properties of the C-dots were retained. No significant difference in the emission properties of samples C, D, and E was observed (Figure S1, Supplementary Materials), indicating that further increasing the C-dot/phospholipid weight ratio percentage is not necessary to improve the emission properties of the micellar nanostructures.

Therefore, the sample labelled as ‘C’, prepared using a C-dot/phospholipid weight ratio percentage of 21%, was demonstrated to retain the optical emission properties and have the smallest average hydrodynamic diameter and highest degree of size uniformity (polydispersity index (PDI) = 0.20 ± 0.02). Considering such findings, this sample was selected for further characterization and experimentation. The rationale behind this choice was that since the typical gaps between mineralized bone fibers (lamellae) are of the order of a few hundred nanometers, only NPs with dimensions falling within the range of 100 to 200 nm can effectively accomplish bone tissue targeting and penetration [40].

The PL QY was assessed for sample C. Due to the processing steps involved and the change in dispersion medium (PBS, 10 mM, pH 7.4), a slight decrease in PL quantum yield (QY = 12.2 ± 0.1%) was observed compared to the PL QY of the ‘as-synthesized’ C-dots (Figure S2B, see Supplementary Materials). A detailed examination of sample C, performed via a transmission electron microscopy (TEM) investigation (inset of Figure 2D), revealed the presence of numerous black spots, each measuring only a few nanometers in size. These dimensions are consistent with the anticipated size of the C-dots and were visibly contained within the round-shaped nanostructures. In the broader view in Figure 2D, the round-shaped nanostructures, attributed to micellar aggregates, exhibit sizes ranging approximately from 40 to 100 nm. Concurrently, the empty micelles appear as smaller nanospheres of approximately 10 nm in diameter. This observation is in good agreement with the findings of the DLS analysis, accounting for the shrinkage of the micellar nanostructures that occurred during the drying process on the grid before the TEM measurements.

**Figure 3:**
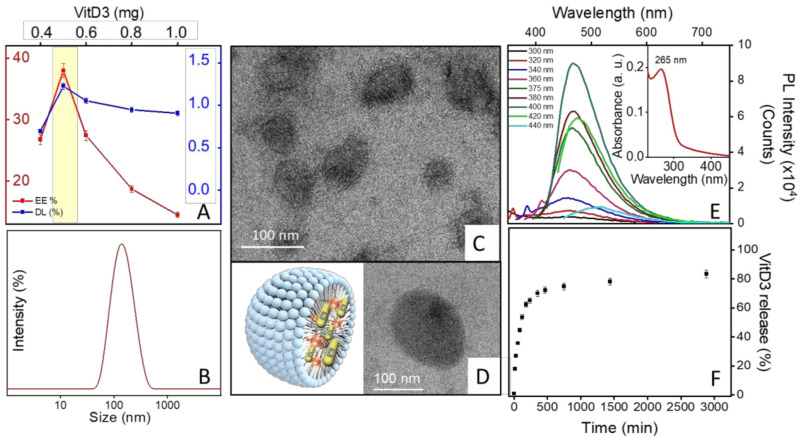
(**A**) Encapsulation efficiency (EE%) and loading (DL%) percentages of the VitD3 for samples of micellar nanostructures co-encapsulating VitD3 and C-Dots, prepared at different starting VitD3/phospholipid weight ratio percentages. (**B**) Size distribution by intensity of the micellar nanostructures co-encapsulating VitD3 and C-Dots obtained by using a VitD3/phospholipid weight ratio percentage of 3.3% and dispersed in PBS (10 mM, pH 7.4). (**C**) representative TEM micrograph along with (**D**) corresponding close-up and schematic sketch. (**E**) PL emission spectra recorded at excitation wavelengths ranging from 300 to 440 nm. Inset: UV-Vis absorbance spectrum. (**F**) In vitro release profile of the VitD3 recorded for the VitD3/C-dot micellar nanostructures (VitD3/phospholipid weight ratio percentage of 3.3%, C-dot/phospholipid weight ratio percentage of 21%).

In the formulation of micellar nanostructures loaded with both VitD3 and C-dots, using a starting C-dot/phospholipid weight ratio percentage of 21%, various nanoformulations were developed by varying the starting amount of VitD3. Specifically, different weight ratio percentages of VitD3 to phospholipids were examined, with values of 2.6%, 3.3%, 4.0%, 5.3%, and 6.6%, corresponding to 0.4 mg, 0.5 mg, 0.6 mg, 0.8 mg, and 1 mg of VitD3, respectively. The actual amount of VitD3 effectively incorporated within the micellar nanostructures was determined via UV-Vis absorption spectroscopy (Figure 3A). This analysis was performed after freeze-drying and subsequent re-dispersing the samples in methanol. The results were expressed as EE% and DL% for each sample, as illustrated in Figure 3A and Figure S3C (see Supplementary Materials). These findings indicate that the maximum values of DL% and EE% were attained when the initial feed of the drug was 0.5 mg. As a result, this specific formulation, selected among those prepared with various initial amounts of VitD3, was chosen for subsequent conjugation with ALE.

The concentration of C-dots incorporated into the formulation, which was prepared at a VitD3/phospholipid weight ratio percentage of 3.3%, was determined to be 37.5 µg/mL. Such an evaluation was carried out by creating a calibration curve based on PL emission spectra under excitation at 375 nm of chloroform dispersion of previously freeze-dried formulations. The PL spectra of the formulations showed the same peculiar emission profiles for the pristine C-dots (Figure 3E), thereby suggesting that C-dots, after their co-encapsulation into the micellar nanostructures with VitD3 (Figure 3B), retain their emission properties, although in this case an additional decrease in their PL QY was detected (7.2 ± 0.2%; Figure S2B, see Supplementary Materials), due to both the further processing and environmental changes that C-dots undergo by passing from the original organic solvent to the hydrophobic micellar cores where they are also co-loaded with VitD3. The absorption spectrum (inset, Figure 3E) of the formulations was dominated by a band at 265 nm attributed to the VitD3 (Figure S3A, see Supplementary Materials) within the micellar system. The absorption features, characteristic of the C-dots, covering the range between 300 and 400 nm (see the spectrum of the ‘as-synthesized’ C-dots inset in Figure 1A as a comparison) were poorly visible, since they fell in the same spectral region of VitD3 absorption or due to the altered chemical environment experienced by the C-dots’ surfaces.

The TEM analysis performed on the VitD3/C-dot micellar nanostructures (Figure 3C,D) revealed the formation of spherical NPs with sizes ranging from 60 nm to 100 nm. The DLS investigations unveiled that the VitD3/C-dot micellar nanostructures exhibited an average hydrodynamic diameter of approximately 130 nm, with a polydispersity index (PDI) of (0.262 ± 0.133). The monomodal size distribution in Figure 3B indicates the complete elimination of empty micelles as a result of the ultracentrifugation process (see experimental section for more details). The ζ-potential value measured for this formulation was (−24.1 ± 3.0) mV, underscoring the good colloidal stability in aqueous solutions and highlighting a negative surface charge, attributed to outer exposed phosphate and carboxylate groups on the surfaces of the micellar structures.

The in vitro release of VitD3 from the micellar nanostructures (sampled at a VitD3/phospholipid weight ratio percentage of 3.3% and C-dot/phospholipid weight ratio percentage of 21%) was monitored using UV-Vis spectroscopy under physiological conditions (PBS 10 mM, pH = 7.4). The release profile in Figure 3F indicates that a payload fraction of about 50% is released into the medium within the first 4 h, followed by more sustained release of vitamin D3, reaching up to (81 ± 3)% at 50 h.

### 2.2. Conjugation with ALE of the Luminescent Micellar VitD3 Containing Nanostructures

**Scheme 1:**
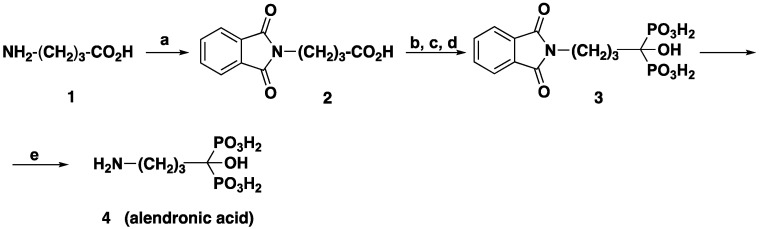
Reaction scheme: (a) phthalic anhydride, triethylamine (Et_3_N_(cat_._)_), toluene, reflux, 5 h; (b) thionyl chloride, reflux, 2 h; (c) tris(trimethylsilyl)phosphite, tetrahydrofuran (THF), 0 °C to r. t., 15 min–15 h; (d) methanol (MeOH), r. t., 1 h; (e) 12 N HCl, reflux, 5.5–20 h.

The ALE was synthesized by reacting the 4-aminobutyric acid (**1**) with phthalic anhydride to afford the *N*-phthaloyl butyric acid (**2**). The *N*-phthaloyl butyric acid (**2**) was converted into (**3**) using a modified Arbuzov’s reaction. Specifically, **2** was first converted into the corresponding acyl chlorides by thionyl chloride and then used as a reagent in the modified Arbuzov’s reaction, in which the phosphorylating agent was the tris(trimethylsilyl)phosphite. The silyl esters of the geminal bisphosphonic acid intermediate were hydrolyzed into compound **3**. The deprotection of the amino group hydrolyzing the phthalimide moiety, achieved with 12 N HCl at reflux, afforded the alendronic acid **4** (Scheme 1) [41,43,44,45,46,47].

ALE was then used for conjugation onto the surfaces of the micellar nanostructures. The conjugation process promoted the formation of covalent bonds between the carboxylic groups on the micellar surface and the amine groups present in the ALE structure. This strategic approach was adopted with the aim of creating nanosystems that have the potential to target bone tissue [38,48].

**Figure 4:**
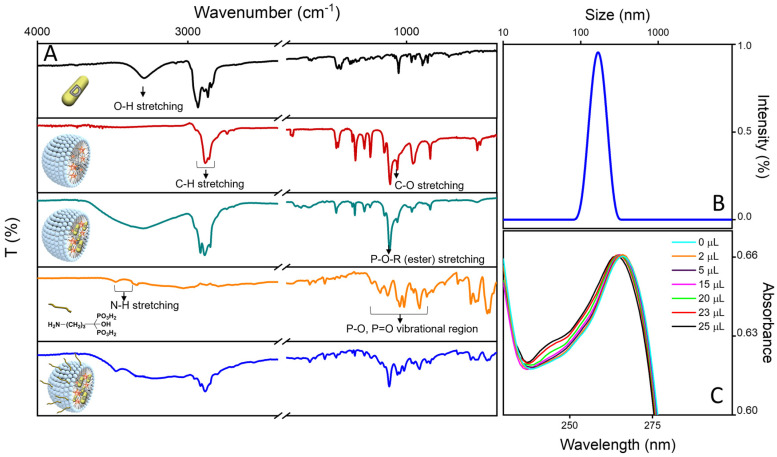
(**A**) FT-IR spectra of free VitD3, luminescent micellar structures and loaded with VitD3, synthesized ALE and ALE conjugated to the surface of the luminescent micellar formulation loaded with VitD3. (**B**) Representative size distribution based on intensity. (**C**) Determination of the ALE concentrations in the micellar formulations based on complexation with Fe(III) ions in acidic conditions.

The effective conjugation of the ALE with the micellar nanoformulations was assessed using an FTIR-ATR (Fourier transform infrared spectroscopy–attenuated total reflection) analysis (Figure 4A). For comparison, the pure VitD3 encapsulated in the nanoformulations and ALE and the bare micellar nanostructures were analyzed. The FTIR spectrum of the luminescent micellar nanostructures loaded with VitD3 revealed two distinct peaks at 3480 and 3350 cm^−1^, corresponding to stretching vibrations of N-H attributable to the primary amine groups within the molecular structure of ALE. In the spectrum of the luminescent micellar nanostructures loaded with VitD3 and conjugated with ALE, a singular N-H peak stretching vibration at 3480 cm^−1^ was evident, indicative of amide bond formation. Additionally, the signal of VitD3 at 3330 cm^−1^, associated with the stretching of the hydrogen bond O-H, and peaks within the 2990 to 2880 cm^−1^ range, attributed to symmetric stretching of aliphatic C-H, were discernible in conjunction with a peak centered at 1110 cm^−1^ arising from C-O-C stretching of the PEG–phospholipid chains within the spectrum of the micellar nanoformulations loaded with VitD3 and conjugated with ALE. A broad band spanning the 1200 to 800 cm^−1^ range, corresponding to phosphate functional groups, was observed in the ALE-conjugated sample due to the overlapping of phosphate esters (1100 cm^−1^) and phosphonate vibrations from lipids and ALE, respectively [49,50]. Consequently, the FTIR-ATR analysis indicated the successful conjugation of ALE onto the micellar surfaces. The spectroscopic investigation of the conjugated sample demonstrated that similarly to previous findings, while the emission properties were retained, a slight decrease in the PL QY (5.2 ± 0.2)% was observed (see Figure S2B, see Supplementary Materials).

DLS analysis performed on the conjugated samples reveals a monomodal size distribution and an increase in the average hydrodynamic diameter that shifts from 130 to about 150 nm (PDI 0.298 ± 0.065) passing from nude micellar to conjugated structures (Figure 4B), while ζ-potential measurements results (−28 ± 4) mV, highlighting relevant colloidal stability in the aqueous solution of the resulting nanoformulations and suggesting that residual negatively charged phosphate and carboxylate groups are still present on the micellar surface.

According to the procedure reported by Kuljanin et al. [46], the ALE conjugated on the surface of the micellar formulations was colorimetrically determined through a complexation reaction with Fe(III) ions, as ALE does not absorb in the UV-Vis spectral region. It is well known that bisphosphonates, including ALE, exhibit strong chelating properties towards metals, including iron, owing to the excellent complexing ability of the O=P-C-P=O moiety, which facilitates the formation of stable metal complexes [46]. The pH condition is a critical parameter for this complex formation, as it heavily depends on the deprotonation degree of the phosphonic acid groups. Since the pKa1 of ALE is approximately 2.43 [51], the complex formation was promoted under highly acidic conditions (pH < 2). The progress of the complex formation was monitored using UV-Vis spectroscopy in the wavelength range of 200 to 300 nm, with the gradual addition of a calibrated amount of an acidic Fe(III) ion solution into an acidic aqueous dispersion containing the ALE-conjugated micellar nanostructures (Figure 4C) [46].

Prior to the introduction of the acidic Fe (III) ion solution, the UV-Vis absorption spectrum of the micellar nanostructures loaded with VitD3 and conjugated with ALE exhibited a band centred at 265 nm, due to the incorporation of the VitD3 in the micellar core (heavenly line, Figure 4C). As the Fe (III) ions were added, the formation of the Fe(III) ion–ALE complex occurred; the absorption band of the complex at 260 nm (Figure S4B, see Supplementary Materials) is hidden by the overlapping strong absorption signal of the VitD3 that falls in the same spectral range. When a new peak emerges at 240 nm (Figure 4C), ascribable to free Fe(III) ions in the acid solution (as shown in the UV-Vis spectrum in Figure S4A, see Supplementary Materials), this indicates that all the ALE molecules on the micellar surface have been complexed [46].

The quantitative evaluation of the amount of ALE covalently conjugated onto the micellar nanostructures resulted in an average concentration of (5.5 ± 0.5) × 10^−4^ M.

### 2.3. Binding Affinity Assay

**Figure 5:**
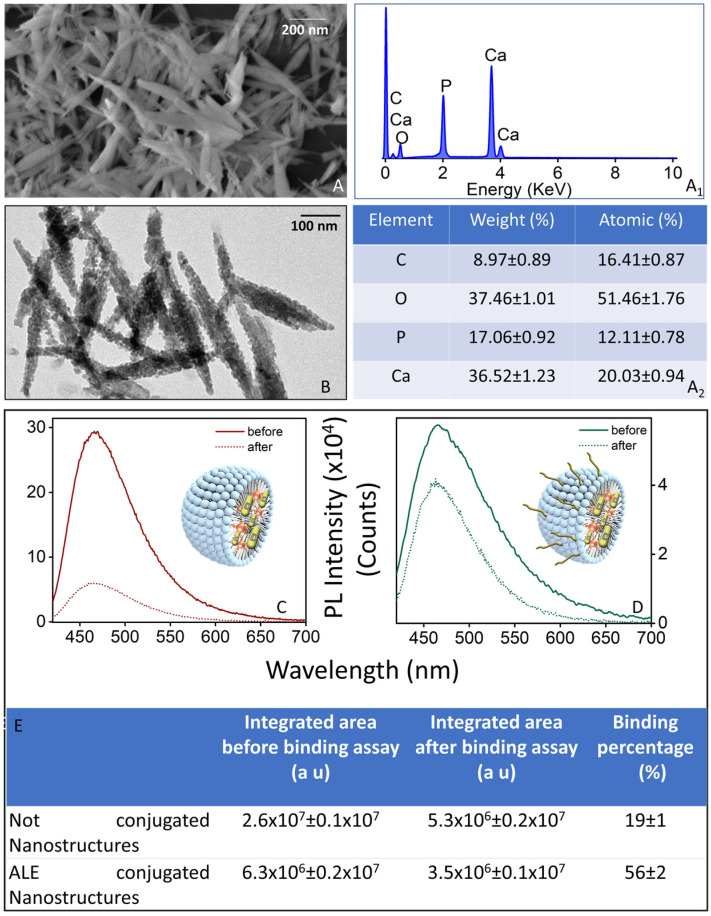
Representative FE-SEM micrograph (**A**), EDX element spectrum (**A1**), element content table (**A2**), and representative TEM micrograph of the HA nanoparticles (**B**). PL spectra, before and after incubation with HA NPs, of the non-conjugated (**C**) and ALE-conjugated (**D**) VitD3/C-dot micellar nanostructures. Table reporting the binding percentage for the non-conjugated and ALE-conjugated samples obtained by estimating the integrated area under the PL spectrum, before and after the incubation of each sample with the HA NPs. The data are presented as means ± S.D (n = 3) (**E**).

HA NPs, synthesized using the method reported in Boanini et al. [33], were used to assess their binding affinity with the ALE-conjugated micellar nanostructures. The rationale behind this evaluation was rooted in the composition of natural bone, wherein 70% of the mineral phase comprises 95% HA. Furthermore, previous studies have proved that BPs, including ALE, exhibit a robust affinity for bone mineral HA [52,53,54,55,56]. Consequently, given this inherent affinity, it is postulated that during the metastatic process, HA may function as a binding receptor for nanosystems conjugated with ALE [57]. The TEM and field emission scanning electron microscopy (FE-SEM) investigation proved the formation of elongated NPs that appeared as needle-like flakes; the statistical analysis based on the TEM investigation (Figure 5A,B) gave an average length of about 58 nm (σ% = 5%).

The chemical composition of the HA NPs derived from the SEM-EDX (energy-dispersive spectroscopy) analysis, reported in Figure 5(A1,A2), provides a Ca/P mole ratio of 1.67 [58], in good accordance with the expected theoretical value [59]. The binding affinity assay was carried out by incubating at 37 °C the luminescent ALE-conjugated VitD3/C-dot micellar nanostructures with the ‘as synthesized’ HA NPs. After 5 h, the HA NPs decorated with the luminescent nanostructures were recovered by centrifugation, re-suspended in PBS, and analyzed by PL spectroscopy (λex = 405 nm). For comparison, the same experimental procedure was used for the non-conjugated VitD3/C-dot micellar nanostructures. By examining the integrated area of the PL spectrum before and after the affinity binding assay for both non-conjugated and ALE-conjugated micellar nanostructures, (Figure 5D), the binding affinity percentages with HA NPs were estimated as approximately 56% and 19% for the ALE-conjugated and non-conjugated samples, respectively. This suggests that a slight non-specific adsorption of the micellar surface on HA NPs concurrently takes place. Nevertheless, the enhanced binding percentage calculated for the ALE-conjugated micellar nanostructures on HA NPs suggests that micellar formulations grafted with ALE could serve as promising candidates for active bone targeting, owing to their favourable affinity for the primary inorganic component of bone.

## 3. Materials and Methods

### 3.1. Materials

The citric acid (anhydrous), ODE (technical grade 90%), HDA (98%), cholecalciferol (Vitamin D3, MW: 384.64 g/mol), ferric chloride hexahydrate (FeCl_3_·6H_2_O), calcium nitrate tetrahydrate (Ca (NO_3_)_2_·4H_2_O), ammonium phosphate ((NH_4_H_2_PO_4_) and sodium hydroxide (NaOH), 4-aminobutyric acid, thionyl chloride (SoCl_2_), and tris(trimethylsilyl) phosphite were purchased from Sigma (St. Louis, MO, USA). The 1,2-dipalmitoyl-sn-glycero-3-phosphoethanolamine-*N*-[methoxy (poly(ethylene glycol))-2000] (16:0 PEG2000 PE, ammonium salt) and 1,2-distearoyl-sn-glycero-3-phosphoethanolamine-*N*-[carboxy(polyethylene glycol)-2000] (DSPE-PEG(2000)–carboxylic acid, sodium salt) were purchased from Avanti Polar Lipids, while the 1-ethyl-3-(3-dimethylaminopropyl)carbodiimide hydrochloride (EDC) and *N*-hydroxylsulfosuccinimide (sulfo-NHS) were purchased from Pierce. The perchloric acid (HClO_4_) was purchased from Fluka. All solvents (Sigma-Aldrich) were of analytical grade. The qqueous solutions were prepared using water obtained from a Milli-Q gradient A-10 system (Millipore, Burlington, MA, USA).

### 3.2. Preparation of PEG–Micellar Nanostructures Loaded with C-Dots and VitD3

The colloidal C-dots were synthesized and purified as previously described [60]. Briefly, the process involved the thermal carbonization of citric acid in the presence of ODE as a high-boiling point solvent and HDA as a surface ligand and nitrogen source. Prior to the synthesis, all reactants were thoroughly dried and degassed and the synthetic process took place under an inert nitrogen atmosphere using a Schlenk line. Here, 1.5 g of HDA was dissolved in 26 mL of ODE under vacuum conditions at 100 °C for 30 min; then, the temperature was raised above the decomposition point of citric acid (153 °C) and 1 g of citric acid was quickly added into the reaction mixture at 200 °C. The reaction proceeded for 3 h before being quenched by cooling to 25 °C. To purify the resulting C-dots, numerous cycles of non-solvent precipitation and re-dispersion in acetone were performed and then the purified C-dots were dispersed in chloroform, ready for further characterization and their encapsulation into micellar nanostructures.

For the preparation of the micellar nanostructures co-encapsulating C-dots and VitD3 (VitD3/C-382 dots/Micelles), 0.15 mL of C-dots (18 mg/mL) and calibrated amounts of VitD3 (1 mg/mL, chloroform solution) were added to the chloroform solution containing PEG-PE and DSPE-PEG-COOH (molar ratio 4:1) at a final volume of 5 mL. Depending on the final concentration of VitD3, the chloroform volumes ranged from 3.7 to 4.3 mL. After the removal of the organic solvent via evaporation, a film composed of VitD3/C-dot/PEG-modified lipids was obtained; then, it was kept under vacuum conditions for 2 h and reconstituted in phosphate-buffered solution (2 mL of 10 mM PBS, pH 7.5). Three cycles of heating (T = 80 °C) and cooling at room temperature were carried out to obtain the VitD3 and C-dot-loaded micellar nanostructures. The samples were purified firstly via centrifugation at 5000× *g* for 1 min to remove the non-encapsulated C-dots or VitD3 and then by using Centricon Plus-70 (membrane Ultracel-PL, 100 kDa, Millipore). Finally, the suspension was ultracentrifugated at 200,000× *g* for 4 h to eliminate the empty micelles that remained suspended while the pellet was recovered and dispersed in PBS. The luminescent micellar nanostructures loaded with C-dots only were prepared by following the same procedure described above, without the addition of the VitD3 to the chloroform solution and omitting the final step of ultracentrifugation.

The quantitative evaluation of the amounts of C-dots actually encapsulated in the lipid nanostructures was performed by freeze-drying the samples (1 mL, aqueous solution) for 24 h at −60 °C (α 1−4 LSC model, CHRIST freeze-dried, Osterode am Harz, Germany) and treating them with 1 mL of chloroform to induce the rupture of the micellar structures and to promote the dispersion of oil-soluble C-dots in the organic solvent. A calibration curve was plotted by performing steady state photoluminescent (PL) measurements (at an excitation wavelength of 375 nm) on the organic solutions containing the phospholipids at the fixed concentration of 7.5 mg/mL and with C-dot concentrations in the range between 21 and 324 µg/mL [38], as reported in Figure S2 (see Supplementary Materials).

### 3.3. Determination of Loading and Encapsulation Efficiency Percentages of Vitamin D3 and In Vitro Release Study

The encapsulation efficiency (EE%) and drug loading (DL%) results were obtained by evaluating the VitD3 contents in the freeze-dried samples. Each sample was treated firstly with chloroform and then, upon solvent evaporation, with methanol to extract and solubilize the VitD3. After centrifugation, the supernatants were characterized using UV-Vis absorbance spectroscopy (PerkinElmer (Waltham, NA, USA) Lambda 20 UV VIS Spectrophotometer). A calibration curve was obtained by recording the maximum absorbance value at 265 nm for standard methanol solutions containing VitD3 in the concentration range of 0.1 to 1 mg/mL, as reported in Figure S3A,B (see Supplementary Materials).

The obtained EE% was as follows:

EE% = (W_t_/W_i_) × 100 (1)

where W_t_ is the total amount of VitD3 in the micellar nanostructures and W_i_ is the total amount of therapeutic compound added as a starting feed in the preparation process.

The DL% is the effective amount of drug (in weight) incorporated in the micellar system, which was calculated as follows:

DL% = (W_t_/W_Mic_) × 100 (2)

where W_Mic_ is the weight of the micellar nanostructures.

A Franz diffusion cell was used to perform the in vitro release study of VitD3 from micellar nanostructures in PBS (10 mM, pH = 7.4) at 37 °C under magnetic stirring. A cellulose acetate membrane with an average pore size of 0.2 µm (Fisher Scientific Milano, Milan, Italy) was placed between the donor and receptor chambers. Here, a 500 µL dispersion of VitD3/C-dot micellar nanostructures in PBS was introduced into the donor chamber and 9 mL of PBS solution was added to the receptor chamber. At scheduled times, 400 µL of receptor solution was taken within 24 h and an equal volume of PBS was added to the receptor chamber. Each collected solution fraction was analyzed using UV-Vis absorbance spectroscopy to evaluate the drug content by using the calibration curve described previously. The in vitro release experiments were carried out in triplicate.

### 3.4. Synthesis of Alendronic Acid

The alendronic acid (4-amino-1-hydroxybutane-1,1-diyl)bis(phosphonic acid (**4** in Scheme 1) [47] was prepared starting from the commercially available 4-aminobutyric acid (**1**), which was allowed to react with phthalic anhydride in toluene under reflux conditions for 5 h, in the presence of a catalytic amount of triethylamine (Et_3_N). Then, the reaction mixture was allowed to reach room temperature and the *N*-phthaloyl butyric (**2**) was extracted with EtOAc and isolated after the solvent was distilled under reduced pressure (79% yield of step (a) in Scheme 1). The *N*-phthaloyl butyric acid (**3,** 2.02 mmol) was solubilized in SOCl_2_ (5.9 mL) and refluxed for 3 h. The solvent was evaporated under reduced pressure, affording a white solid, which in turn was dissolved in dry tetrahydrofuran (THF) (4 mL) at 0 °C before the addition of tris(trimethylsilyl)phosphite (2.02 mL, 6.06 mmol) for 40 min. Then, the mixture was stirred for 16 h at room temperature. The methanol (MeOH) (3 mL) was added and the resulting solution was stirred for 5 h at room temperature. The solvent was evaporated under vacuum conditions and the white solid was washed three times with a toluene/hexane mixture (1:1) (10 mL), then with diethyl ether and dried under vacuum, obtaining intermediate **3** (98% yield of steps (b), (c), and (d) in Scheme 1). A solution of **3** (2.01 mmol) in 12 N HCl (17 mL) was refluxed for 22 h. The solvent was removed under reduced pressure, obtaining a white solid that was washed with ethanol (70 mL) at 70 °C. The alendronic acid (**4**) solid was then isolated via filtration of the solution, washed with warm ethanol, and dried under vacuum conditions (74% yield of step (e) in Scheme 1). 1H NMR (500 MHz, D2O/NaOD, δppm): 1.32–1.36 (m, 2H), 1.47–1.50 (m, 2H), 2.22 (t, J = 7.5 Hz, 2H). 31P NMR (202 MHz, D2O/NaOD, δppm): 18.96 (s, 2P) (1H and 31P NMR spectra are reported in Figure S5 of the Supplementary Materials).

### 3.5. Alendronate Bioconjugation of the Micellar Nanostructures Loaded with C-Dots and Vitamin D3

The alendronate sodium trihydrate (4-amino-1-hydroxy-1-phosphonobutyl phosphonic acid, monosodium, ALE) was synthesized as previously reported [47]. The carboxylic groups on the micellar surface were activated in PBS buffer (10 mM, pH = 7.4) using sulfo-NHS/EDC at a molar ratio of 1:10 for 15 min at 25 °C. Then, after removing the sulfo-NHS/EDC excess using Centricon Plus-70 (100 kDa, 5000× *g*, 10′, Millipore), the ALE was added to the aqueous dispersion containing the amine-reactive micellar nanostructures at a final concentration of 1.4 × 10^−3^ M and left to react overnight (25 °C). Subsequently, the ALE-conjugated lipid nanostructures were purified using Centricon Plus-70 and washed with PBS buffer to remove unreacted cross-linking molecules. For the evaluation of the ALE content conjugated on the micellar surface, an assay based on the formation of a chromophore complex between ALE and iron (III) ions was performed, according to the procedure reported by Kuljanin et al. [46]. Briefly, an aqueous dispersion of the ALE-conjugated lipid nanostructures was diluted with an aqueous solution of perchloric acid (2 N, pH < 2). Subsequently, a solution of FeCl_3_ 10^−2^ M in HClO_4_ (0.2 N) was added dropwise until the formation of the complex was complete. After each addition, the formation of the ALE/iron complex with absorption at λ = 260 nm was monitored using UV-Vis spectroscopy.

### 3.6. Binding Assay to Synthetic Hydroxyapatite

The HA NPs were synthesized by mixing a calibrated amount of 10 mL of 100 mM Ca(NO_3_)_2_·4H_2_O solution and 10 mL of 65 mM NH_4_H_2_PO_4_ (pH = 10), as reported by Boanini et al. [33]. In particular, 10 mL of Ca(NO_3_)_2_·4H_2_O aqueous solution was heated at 90 °C and the NH_4_H_2_PO_4_ aqueous solution was added dropwise under stirring. The mixture (20 mL, final volume) was allowed to react for 5 h, then the pellet recovered via centrifugation and dispersed in PBS.

To evaluate the binding affinity of the micellar nanostructures to bone, VitD3/C-dots/micelles (11 mg/mL), used as the control, and VitD3/C-dots/ALE–micelles (11 mg/mL) were incubated with HA NPs (500 µL, 0.6 mg/mL) at 37 °C for 1 h. Then, the incubated samples were centrifugated and the pellet was dispersed in 500 µL of PBS. These solutions were analyzed via steady-state PL measurements.

### 3.7. DLS Investigation and ζ-Potential Measurements

A Zetasizer Nano ZS (Malvern Instruments Ltd., Worcestershires, UK) system was used to evaluate the mean hydrodynamic diameter, polydispersity index (PDI), and ζ-potential values for all of the samples, which were dispersed in PBS buffer (10 mM pH 7.4), as previously reported [61].

### 3.8. TEM and SEM Investigation

The TEM analysis was performed using a Jeol TEM-1011 microscope, provided with an Olympus Quemesa Camera (11 Mpx, Olympus, Tokyo, Japan). The samples were prepared by depositing on a carbon-coated Cu grid (400 mesh) a drop (5 μL) of C-dots or the micellar nanostructure solution. For sample staining, the grid was placed on the top of a drop of an aqueous phosphotungstic acid solution 2% (*w*/*v*) for 30 s, after the evaporation solvent. Then, the grid was washed with ultrapure water and left to dry. A statistical size analysis was performed on the C-dots samples using the Image J 1.53e analysis program.

Field emission scanning electron microscopy (FE-SEM) was performed using a Zeiss (Oberkochen, Germany) Sigma microscope operating in the range of 0.5–20 kV and equipped with an in-lens secondary electron detector and an INCA energy-dispersive spectroscopy (EDS) detector. The HA spray-dried samples were mounted onto stainless steel sample holders using double-sided carbon tape and grounded by silver paste.

### 3.9. FTIR-ATR Characterizazion

The FTIR characterization was carried out using a 670 FTIR spectrometer (Varian, Palo Alto, CA, USA) with a diamond ATR accessory measuring 2 mm and a deuterated molecules of tryglicine sulfate (DTGS) detector. Five microliters (5 µL) of each sample were placed on the internal reflection element and the solvent was allowed to evaporate. The spectra were recorded in the range of 4000–400 cm^−1^, acquiring 16 scans with a nominal resolution of 1 cm^−1^.

### 3.10. Photophysical Investigation

A Cary 5000 (Varian) UV-Vis/NIR spectrophotometer and a Fluorolog 3 (HORIBA Jobin-Yvon) spectrofluorometer, equipped with a time-correlated single photon counter (TCSPC), were used to record the UV-Vis absorption and PL emission spectra, respectively. A “Quanta-phi” (HORIBA Jobin-Yvon, Palaiseau, France) integrated sphere coated by Spectralon was used for the evaluation of the PL emission and absolute quantum yield (QY) of the C-dots dispersed in the organic solvent and of the luminescent micellar nanostructures dispersed in aqueous solution at the excitation wavelength of 360 nm.

## 4. Conclusions

Novel, optically traceable, PEG-stabilized micellar nanostructures were successfully designed and fabricated for the targeted delivery of VitD3. A comprehensive optical and morphological characterization process substantiated the relevant colloidal stability of these innovative nanoformulations in aqueous solutions, with an average diameter measuring less than 200 nm. The concentration of ALE conjugated on the micellar surfaces was quantified using a method reliant on the formation of a chromophoric Fe(III) ion–ALE complex. The optical emission properties of the C-dots were still preserved after their incorporation into the hydrophobic micellar core, suggesting that the obtained optically traceable nanostructures can act as luminescent probes if exploited for in vitro studies. Furthermore, the HA NPs were synthesized and characterized in terms of their morphology and chemical composition. These HA NPs were used to assess the potential ability of the ALE-conjugated nanostructures towards the primary component of the rigid bone structure. The overall findings underscore the efficacy of the mentioned nanoformulations as promising and novel optically traceable candidates for their use in in vitro studies, serving in the initial stages for validation in achieving efficient active bone targeting. Ultimately, this could pave the way for their prospective application in addressing SREs associated with cancer metastases in the future.

## Data Availability

Data are contained within the article and Supplementary Materials.

## Supplementary Materials

The following supporting information can be downloaded at https://www.mdpi.com/article/10.3390/molecules29102367/s1. Figure S1. PL emission spectra of micellar nanostructures prepared at different starting C-dot/PEG–phospholipid weight ratio percentages. Figure S2. Calibration curve of C-dots assessed using PL spectroscopy (λex 360 nm) and the absolute QY (%) of C-dots in CHCl_3_ dispersion and C-dots/micelles, VitD/C-dots/micelles, and VitD/C-dots/ALE–micelles nanoformulations redispersed in CHCl_3_. Figure S3. UV-Vis absorption spectrum of the VitD3 and the calibration curve of the VitD3. Figure S4. UV-Vis absorption spectrum of Fe^3+^ and synthesized ALE-Fe^3+^ complex. Figure S5. The 1H and 31P NMR spectra of the target alendronate.
