# Supplementary material for: Luminescent Alendronic Acid-Conjugated Micellar Nanostructures for Potential Application in the Bone-Targeted Delivery of Cholecalciferol
Source: Molecules. 2024 May 17;29(10):2367. doi: 10.3390/molecules29102367 (PMC11123821; doi:10.3390/molecules29102367)
Supplement: Supplementary file 1 [file molecules-29-02367-s001.zip › molecules-2974168-supplementary.pdf]

molecules

Supplementary Material

Luminescent Alendronic Acid-Conjugated Micellar Nanostructures for Potential Application in the Bone-Targeted Delivery of Cholecalciferol

Federica Rizzi [1,2], Annamaria Panniello [1], Roberto Comparelli [1,2], Ilaria Arduino [3], Elisabetta Fanizza [1,2,4], Rosa Maria Iacobazzi [3], Maria Grazia Perrone [5], Marinella Striccoli [1], Maria Lucia Curri [1,2,4], Antonio Scilimati [5], Nunzio Denora [3,*] and Nicoletta Depalo [1,2,*]

1 CNR-Institute for Chemical and Physical Process, 70125 Bari, Italy; f.rizzi@ba.ipcf.cnr.it (F.R.); a.panniello@ba.ipcf.cnr.it (A.P.); r.comparelli@ba.ipcf.cnr.it (R.C.); elisabetta.fanizza@uniba.it (E.F.); m.striccoli@ba.ipcf.cnr.it (M.S.); marialucia.curri@uniba.it (M.L.C.)
2 Consorzio Interuniversitario Nazionale per la Scienza e Tecnologia dei Materiali (INSTM), 50121 Firenze, Italy
3 Department of Pharmacy—Pharmaceutical Sciences, University of Bari, 70125 Bari, Italy; ilaria.arduino@uniba.it (I.A.); rosa.iacobazzi@uniba.it (R.M.I.)
4 Department of Chemistry, University of Bari, 70125 Bari, Italy
5 Research Laboratory for Woman and Child Health, Department of Pharmacy—Pharmaceutical Sciences, University of Bari, 70125, Bari, Italy; mariagrazia.perrone@uniba.it (M.G.P.); antonio.scilimati@uniba.it (A.S.)
* Correspondence: nunzio.denora@uniba.it (N.D.); n.depalo@ba.ipcf.cnr.it (N.D.)

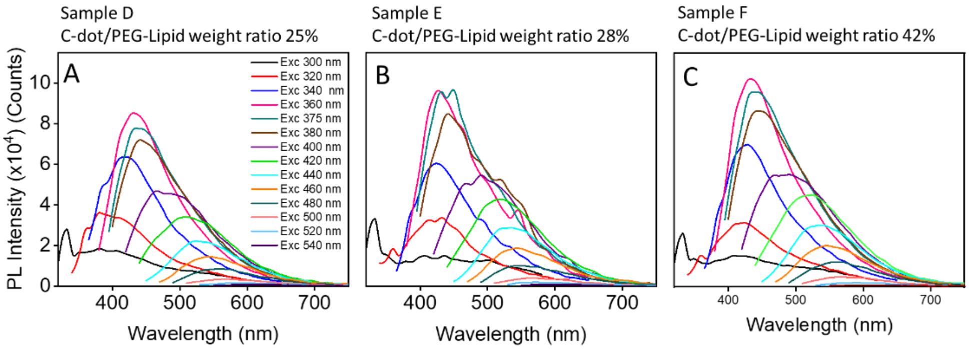

Figure S1. PL emission spectra recorded at the excitation wavelength from 300 to 540 nm of micellar nanostructures prepared at different starting C-dot/PEG-phospholipid weight ratio percentage: A) 25%, B) 28% and C) 42% that correspond to the samples D, E and F of the table in the Figure 2A of the manuscript. PL emission spectra of the samples recorded in PBS buffer (1:3 dilution).

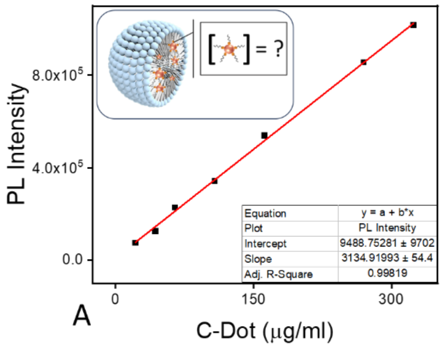

B

Sample	QY(%)@360nm
C-Dot	25.3±0.1
C-Dot/micelles	12.2±0.1
VitD/C-Dot/micelles	7.2±0.2
VitD/C-Dot/micelles+ALE	5.2±0.2

Figure S2. A) Calibration curve of C-dots by PL spectroscopy (λex 360 nm) obtained starting from the concentration of C-dots from 21 to 324 μg/mL, added to a solution containing the phospholipids at the fixed concentration of 7.5 mg/mL; B) Absolute QY (%) of C-dots in $CHCl_3$ dispersion and C-dots/micelles, VitD/C-dots/micelles and VitD/C-dots/micelles-ALE nanoformulations redispersed in $CHCl_3$ as a function of the excitation wavelength (360 nm).

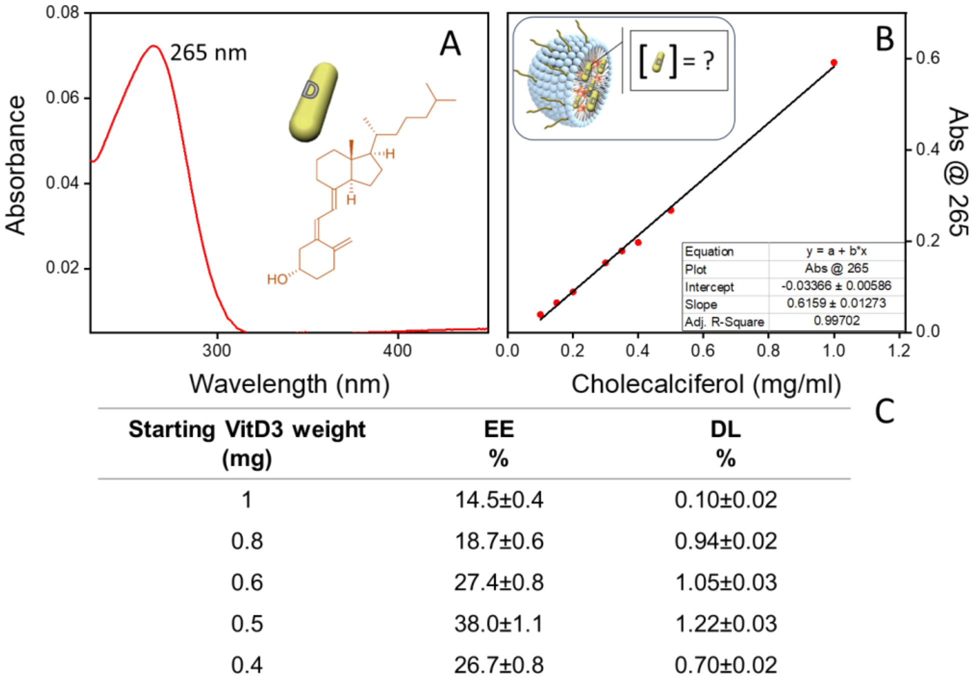

C

Starting VitD3 weight (mg)	EE %	DL %
1	14.5±0.4	0.10±0.02
0.8	18.7±0.6	0.94±0.02
0.6	27.4±0.8	1.05±0.03
0.5	38.0±1.1	1.22±0.03
0.4	26.7±0.8	0.70±0.02

Figure S3. A) UV-Vis absorption spectrum of the VitD3 in methanol; B) Calibration curve of VitD3 plotted by preparing standard methanol solutions containing VitD3 in the concentration range of 0.1 and 1 mg/mL, obtained by recording the maximum absorbance value of each solution at 265 nm; C. EE% and DL% values of different nanoformulations obtained starting from different initial amounts of VitD3.

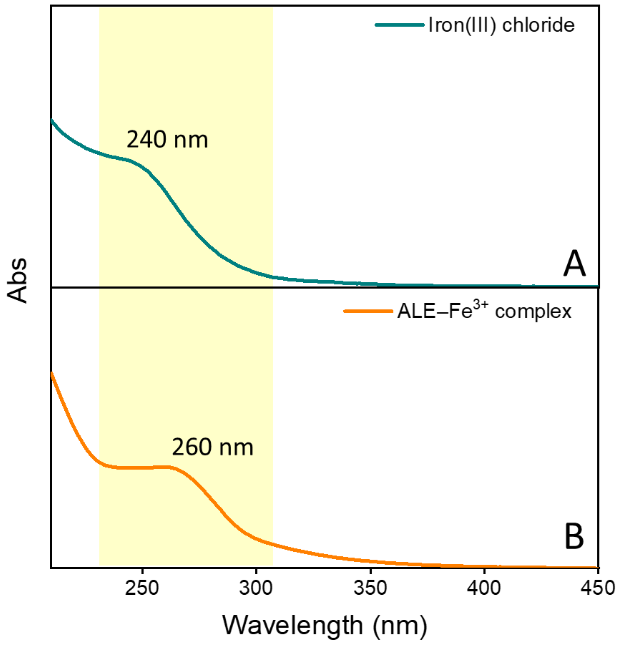

Figure S4. UV-Vis absorption spectrum of A) $Fe^{3+}$and B) synthesized ALE-Fe^{3+}complex.

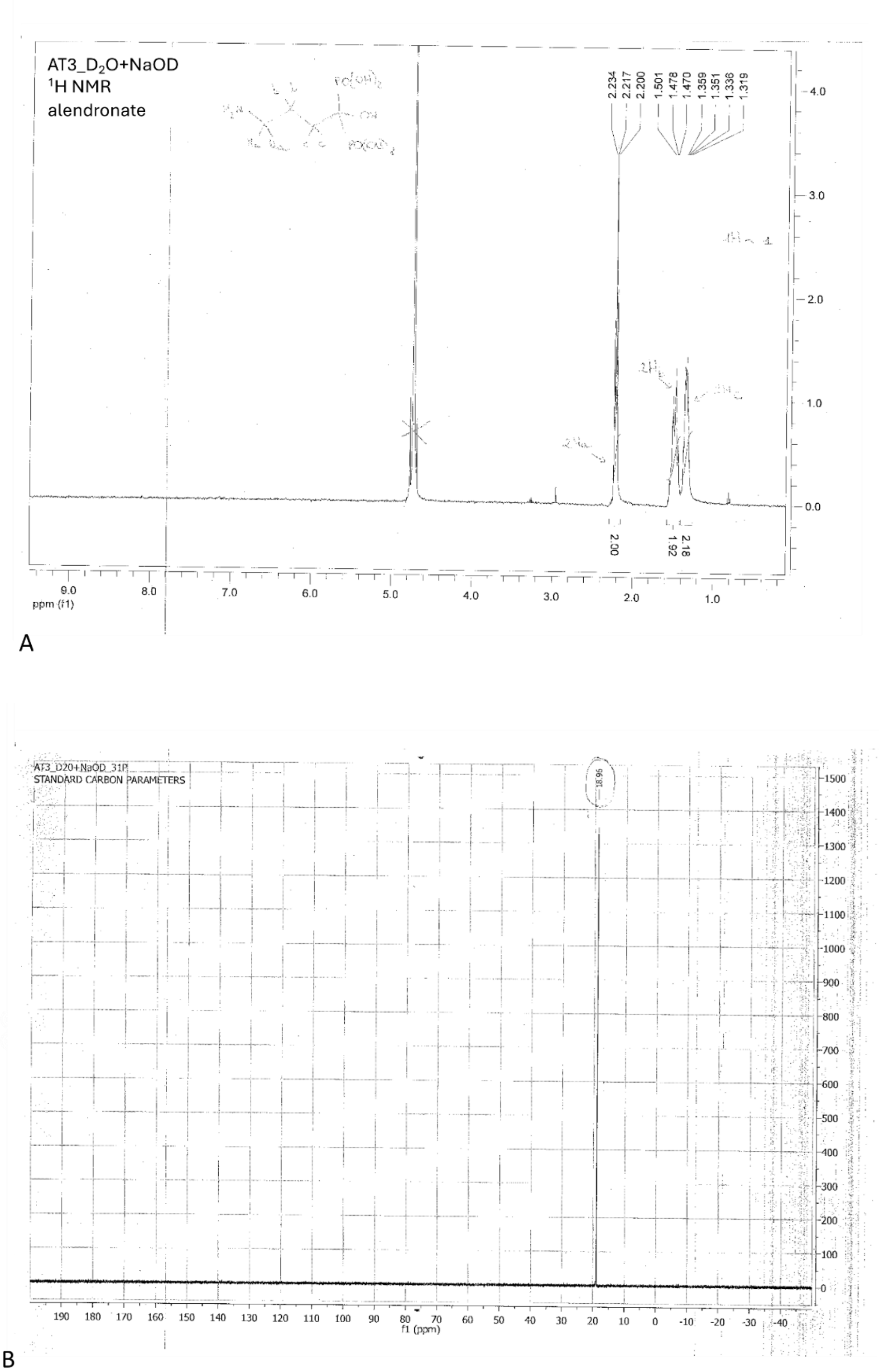

Figure S5. ^{1}H and ^{31}P NMR spectra of the target alendronate: A) Alendronate ^{1}H NMR spectrum recorded in D_2O-NaOD; B) Alendronate ^{31}P NMR spectrum recorded in D_2O-NaOD.
